# Associations of 35 Serum and Urine Biomarkers With Vascular Dementia—A Mendelian Randomization Study

**DOI:** 10.1002/brb3.70736

**Published:** 2025-10-20

**Authors:** Xiaomin Zhu, Yanan Hu, Jieshuang Lin, Guifeng Zhuo, Yingrui Huang, Yulan Fu, Ying Zhang, Lin Wu, Wei Chen

**Affiliations:** ^1^ Department of Neurology The First Affiliated Hospital of Guangxi University of Chinese Medicine Nanning China; ^2^ The First Clinical College of Medicine Guangxi University of Chinese Medicine Nanning China; ^3^ Ziwei comunity health care service center, The Second Affiliated Hospital Cuhk‐Shenzhen Longgang District People's Hospital of Shenzhen Shenzhen China; ^4^ School of Chinese Medicine Hong Kong Baptist University Hong Kong China; ^5^ Scientific Laboratorial Centre Guangxi University of Chinese Medicine Nanning China

**Keywords:** biomarkers, causal relationship, Mendelian randomization, vascular dementia

## Abstract

**Background:**

The relationship between serum and urine biomarkers with vascular dementia (VD) has been increasingly highlighted by observational studies. Yet, the causal nature underlying these associations remains elusive.

**Objective:**

This research seeks to elucidate the causal relationships between 35 prevalent serum and urine biomarkers and the risk of VD onset through Mendelian randomization methods.

**Methods:**

This study employs bidirectional Mendelian randomization, incorporating both forward and reverse approaches, to examine these potential causal links. The findings from the Mendelian randomization are further analyzed for pleiotropy, heterogeneity, and sensitivity to ensure robustness.

**Results:**

The analysis through forward Mendelian randomization reveals that elevated levels of aspartate aminotransferase (AST) and triglycerides (TG) are linked to an elevated risk of developing VD, whereas higher levels of direct bilirubin (DBil) appear to mitigate this risk. These outcomes are corroborated by subsequent analyses for pleiotropy, heterogeneity, and sensitivity. On the other hand, reverse Mendelian randomization indicates that VD does not causally influence the levels of the 35 examined serum and urine biomarkers.

**Conclusion:**

The outcomes of this Mendelian randomization study affirm the causal influence of biomarkers AST, TG, and DBil on the progression of VD. These insights pave the way for leveraging AST, TG, and DBil as biochemical indicators for the forecasting, screening, and early diagnosis of VD, offering avenues for targeted interventions and management.

## Graphical abstract

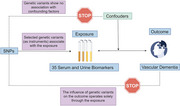

**Abbreviations**
AST: aspartate aminotransferase
DBil: direct bilirubin
TG: triglycerides
VD: vascular dementia

## 1 Introduction

Vascular dementia (VD) emerges as a degenerative vascular disorder affecting the brain, caused by the blockage of blood supply to the brain, and manifested by a decline in memory and cognitive functions (Shen et al. 2021). Recognized as the second most prevalent subtype of dementia, VD constitutes approximately 20% of all dementia cases (Bir et al. 2021). The advancing age of the global population is leading to an increased prevalence of VD. This trend adversely affects patients' quality of life and imposes substantial economic burdens on both families and societal structures (Sontheimer et al. 2021). The current pharmacological treatments for VD present notable limitations, underscoring the critical need for exploring the molecular mechanisms underlying VD and developing innovative strategies for its prevention and treatment.

The onset of VD involves a complex and chronic interplay of pathological mechanisms. At its core, VD pathology is driven by prolonged cerebral underperfusion, resulting in a shortage of essential nutrients like oxygen, glucose, and amino acids. This shortage can cause damage or necrosis in central nervous system tissues and foster the progression of neuroinflammatory processes (Han et al. 2020). The routine assessment of serum and urine biomarkers plays a pivotal role in diagnosing and monitoring various chronic conditions. Gaining insights into the levels of specific biomarkers can significantly influence the early detection, therapeutic intervention, and prognostic evaluation of vascular dementia. A recent initiative by the UK Biobank (UKB) involved the laboratory analysis of 35 common biomarkers in serum and urine, assessing their potential link to the risks of chronic kidney disease, type‐2 diabetes, and gout (Sinnott‐Armstrong et al. 2021). Nonetheless, the causal relationships between these biomarkers and VD remain unexplored. Consequently, additional research is essential to elucidate the causal connection between common biomarkers in serum and urine and the risk of VD deficiency.

Leveraging Mendelian randomization (MR), anchored in genome‐wide association studies (GWASs), offers a robust epidemiological tool for this investigation. MR utilizes single nucleotide polymorphisms (SNPs) as instrumental variables (IVs) to evaluate the causal effects of exposure factors on outcomes. This study thus embarks on both forward and reverse MR analyses to decipher the causal links between serum and urine biomarkers and the susceptibility to VD.

## 2 Materials and Methods

### 2.1 Data Source and Instrumental Variables

We gathered summary statistics for 35 blood and urine biomarkers from existing literature (Sinnott‐Armstrong et al. 2021), involving 363,228 participants from the UKB. The GWAS summary data for VD were obtained from the FinnGen database (https://www.finngen.fi/en); demographic details: https://r10.risteys.finregistry.fi/endpoints/F5_VASCDEM. The analysis incorporated five ICD‐10‐coded subtypes: sudden onset (F01.0), mixed, multiple infarctions(F01.1), subcortical (F01.2), and unspecified (F01.9). European‐exclusive genetic data from independent sources were utilized to satisfy core assumptions of two‐sample MR, minimizing sample overlap effects. Covariate adjustments included age, sex, population structure, genotyping batch, and 10 principal components. The R10 version of this dataset included data on 2717 VD cases and 393,024 controls. All summary data referenced in this study are openly available and can be freely downloaded. Ethical approval was secured for each GWAS included in our analysis.

For a more thorough analysis, SNPs closely linked with protease activity (*p* < 5.0×10^−8^, a threshold adapted to fit the constraints of sample size) were selected as IVs. In the absence of genome‐wide significant SNPs for use as IVs, SNPs with significance levels below the genome‐wide threshold (*p* < 5 × 10^−6^) were considered as potential IVs. Linkage disequilibrium (LD) analyses were performed using genomic sample data, with parameters set to *r* ^2^< 0.001 within a 10,000 kb radius. The robustness of the IVs was quantified by computing the *F*‐statistic, where an *F* > 10 indicated a robust instrument free of weak instrument bias, and IVs with an *F* < 10 were discarded.

### 2.2 Study Design

**FIGURE 1:**
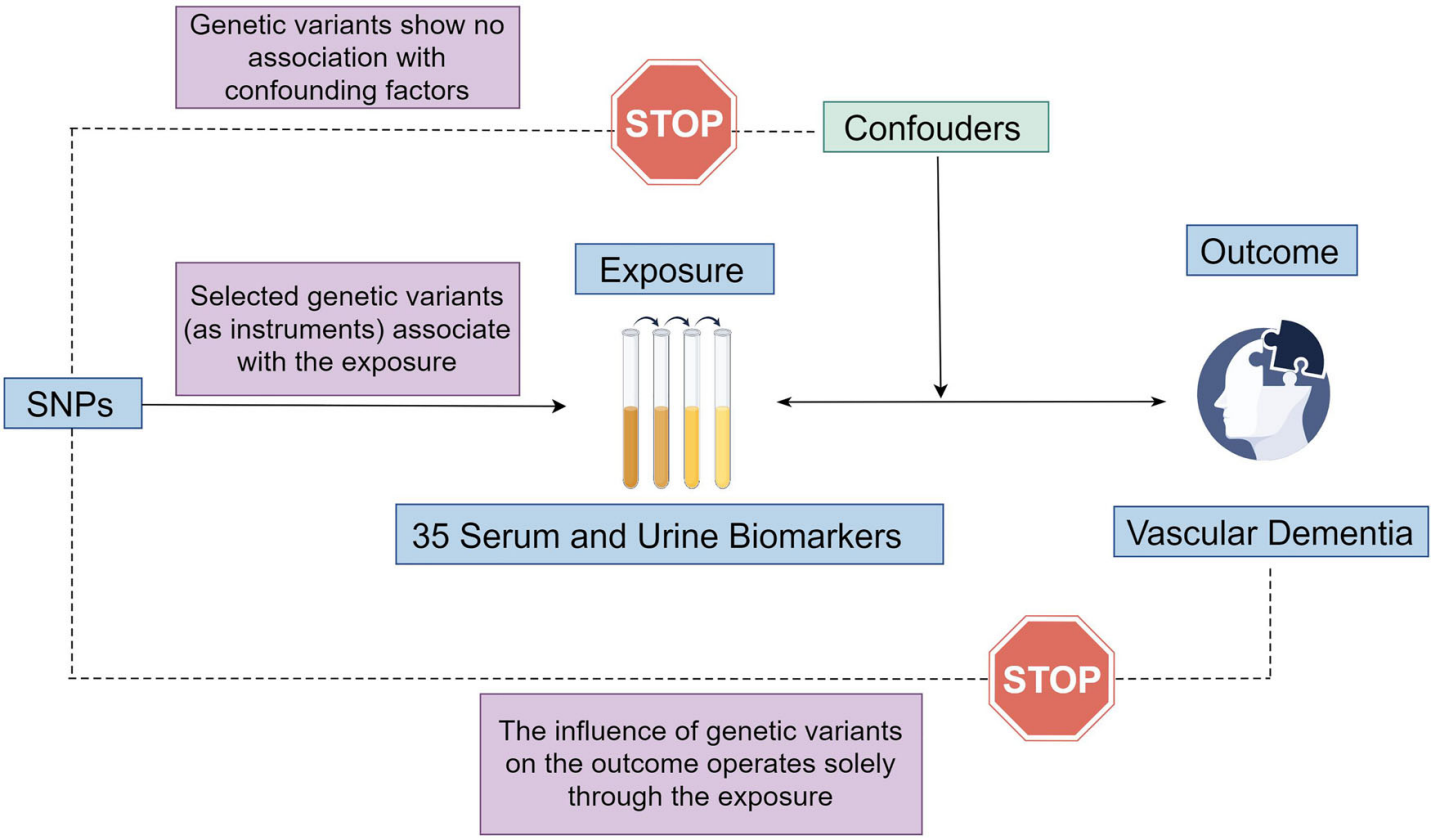
Flowchart illustrating the framework for Mendelian randomization (MR) analysis concerning 35 serum and urine biomarkers and vascular dementia.

An overview of the analysis is shown in Figure 1. The investigation commenced with a forward MR analysis, treating the 35 blood and urine biomarkers as the exposures and VD as the outcome. This was followed by a reverse MR analysis, with VD as the exposure and the biomarkers as outcomes, to assess reverse causality. The same GWAS datasets mentioned previously were employed for these analyses. Our research adhered to the STROBE‐MR guidelines, ensuring all methods were conducted in alignment with these standards (Skrivankova et al. 2021). The original studies involved in this research had already obtained ethical approval, and no additional informed consent or ethical approval was required.

### 2.3 Statistical Analysis

This research applied two‐sample MR analysis techniques including the inverse variance‐weighted (IVW) method, weighted median, MR‐Egger regression, simple mode, and weighted mode to deduce causal relationships (Bowden et al. 2015, 2016). The IVW method was the principal technique for estimating aggregate effect sizes. It synthesizes the MR effect estimates from individual SNPs to provide an aggregate weighted estimate of potential causal effects, proving most accurate in the absence of pleiotropy among IVs (Huang et al. 2021). MR analyses were performed using the R TwoSampleMR package.

Cochran's *Q* test was utilized to evaluate heterogeneity among SNPs (Greco et al. 2015). A fixed‐effect model was applied if no significant heterogeneity was observed (*p* > 0.05); otherwise, a random‐effects model was implemented. Pleiotropy was assessed using the MR‐Egger intercept (with a significance intercept *p* value < 0.05) (Burgess and Thompson 2017). Additionally, the MR‐PRESSO method was employed to identify and remove potential outliers, followed by re‐analysis. A leave‐one‐out sensitivity analysis further scrutinized the impact of individual SNPs on the causal inference. All statistical analyses were conducted in R software (version 4.3.0).

## 3 Results

### 3.1 Causal Effects of 35 Blood and Urine Biomarkers on VD

Adhering to the IV selection criteria, the biomarkers Apolipoprotein A (ApoA), Apolipoprotein B (ApoB) adjstatins, AST, C reactive protein, DBil, and TG were represented by 2361, 2455, 2374, 2336, 2588, and 2328 SNPs, respectively. The *F*‐statistics for these SNPs all exceeded 10, suggesting a low risk of bias due to weak IVs in this study.

In the forward MR analysis involving these 35 blood and urine biomarkers in relation to VD, no significant heterogeneity was found among the IVs for DBil (*p*>0.05), suggesting the suitability of a fixed‐effect model. Conversely, some degree of heterogeneity was detected among the IVs for AST and TG (*p* < 0.05), leading to the application of a random‐effects model. The MR‐Egger regression intercepts for AST, DBil, and TG were virtually zero, signifying no presence of horizontal pleiotropy (*p* > 0.05). However, the tests for pleiotropy in ApoA, ApoB adjstatins, and C reactive protein indicated horizontal pleiotropy among their IVs (*p* < 0.05), rendering the MR analysis results for these biomarkers as unreliable. For detailed outcomes, see Table 1.

**TABLE 1:** Testing for heterogeneity and horizontal pleiotropy.

Exposure—blood and urine biomarkers	MR‐Egger intercept		MR Egger			Inverse variance weighted		
	Egger intercept	*p* value	*Q*	*Q*_df	*Q_p*val	*Q*	*Q*_df	*Q_p*val
Apolipoprotein A	0.005	0.016	1045.977	816.000	0.000	1053.378	817.000	0.000
Apolipoprotein B adjstatins	−0.004	0.043	935.332	781.000	0.000	940.274	782.000	0.000
Aspartate aminotransferase	0.001	0.636	936.742	771.000	0.000	937.015	772.000	0.000
C reactive protein	0.010	0.000	1011.630	809.000	0.000	1038.082	810.000	0.000
Direct bilirubin	−0.001	0.619	739.510	680.000	0.056	739.779	681.000	0.059
Triglycerides	0.001	0.510	1080.469	860.000	0.000	1081.014	861.000	0.000

**FIGURE 2:**
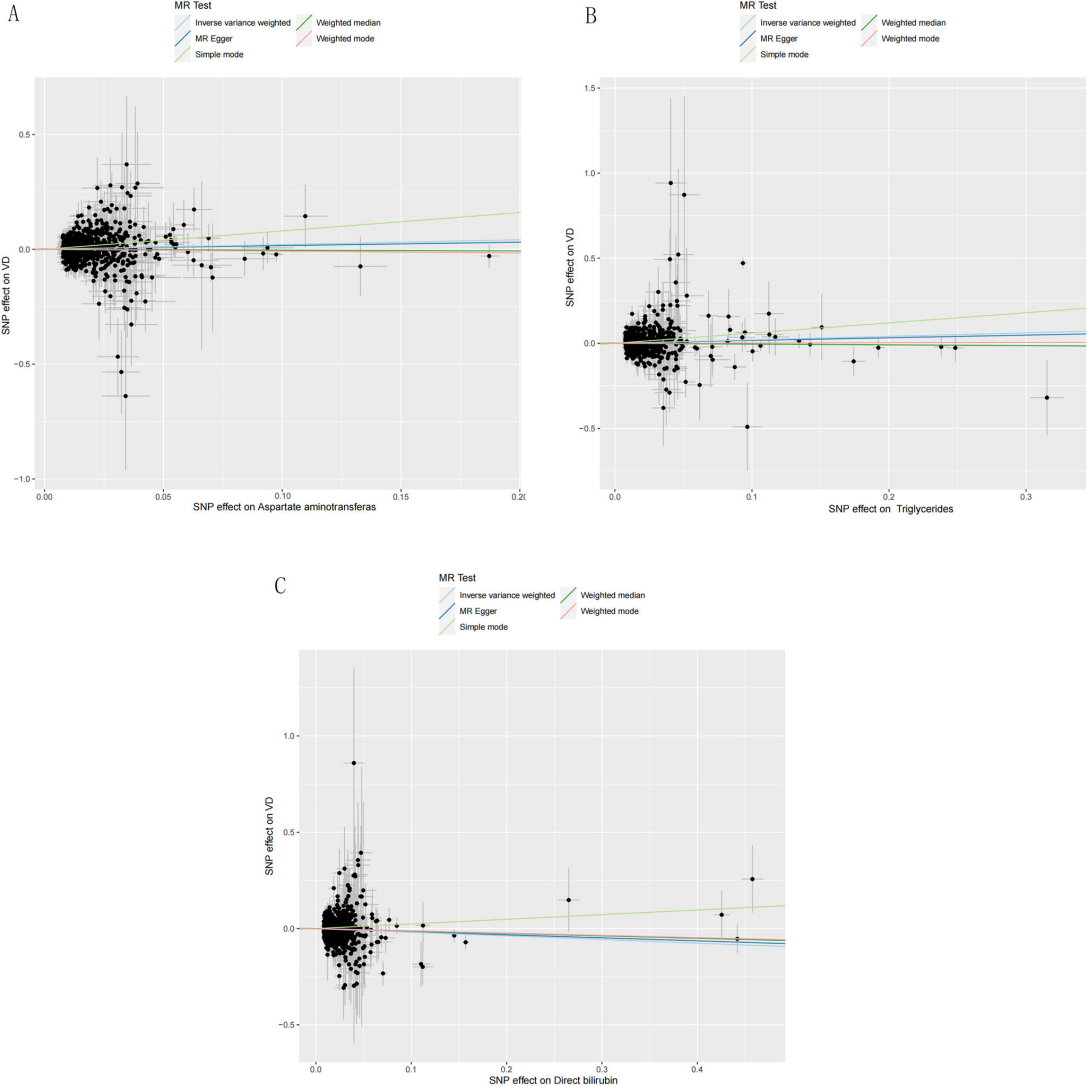
Scatter plot of the association between AST (A), TG (B), and DBil (C) on VD.

Given the absence of horizontal pleiotropy among the IVs for AST, DBil, and TG, the IVW method was employed for the primary MR analysis. The analysis demonstrated a causal association between an increased risk of VD and higher levels of AST (OR = 1.230, CI: 1.053–1.436, *p* < 0.05) and TG (OR = 1.227, CI: 1.079–1.396, *p* < 0.05). On the other hand, an elevated level of DBil (OR = 0.828, CI: 0.721–0.951, *p* < 0.05) was found to be causally associated with a reduced risk of VD. For more insights, refer to Figure 2 and Table 2.

**TABLE 2:** Mendelian randomization analysis of the main results.

Exposure—blood and urine biomarkers	MR Egger		Weighted median		Inverse variance weighted		Simple mode		Weighted mode	
	OR (95%CI)	*p* value	OR (95%CI)	*p* value	OR (95%CI)	*p* value	OR (95%CI)	*p* value	OR (95%CI)	*p* value
Apolipoprotein A	0.734(0.605–0.890)	0.002	0.832(0.664–1.043)	0.111	0.878(0.774–0.996)	0.043	0.731(0.409–1.308)	0.292	0.884(0.710–1.099)	0.267
Apolipoprotein B adjstatins	1.262(1.080–1.474)	0.003	1.159(0.954–1.407)	0.138	1.130(1.009–1.266)	0.035	1.053(0.643–1.723)	0.838	1.107(0.942–1.301)	0.218
Aspartate aminotransferase	1.161(0.873–1.543)	0.304	0.965(0.725–1.285)	0.807	1.230(1.053–1.436)	0.009	2.226(1.158–4.279)	0.017	0.924(0.674–1.266)	0.622
C reactive protein	0.540(0.434–0.672)	0.000	0.831(0.627–1.102)	0.199	0.801(0.695–0.922)	0.002	0.926(0.428–2.008)	0.847	1.016(0.656–1.575)	0.943
Direct bilirubin	0.855(0.708–1.034)	0.106	0.881(0.678–1.145)	0.344	0.828(0.721–0.951)	0.007	1.273(0.576–2.812)	0.551	0.889(0.738–1.070)	0.214
Triglycerides	1.169(0.962–1.420)	0.117	0.956(0.775–1.179)	0.673	1.227(1.079–1.396)	0.002	1.822(1.063–3.124)	0.029	1.015(0.830–1.240)	0.887

**FIGURE 3:**
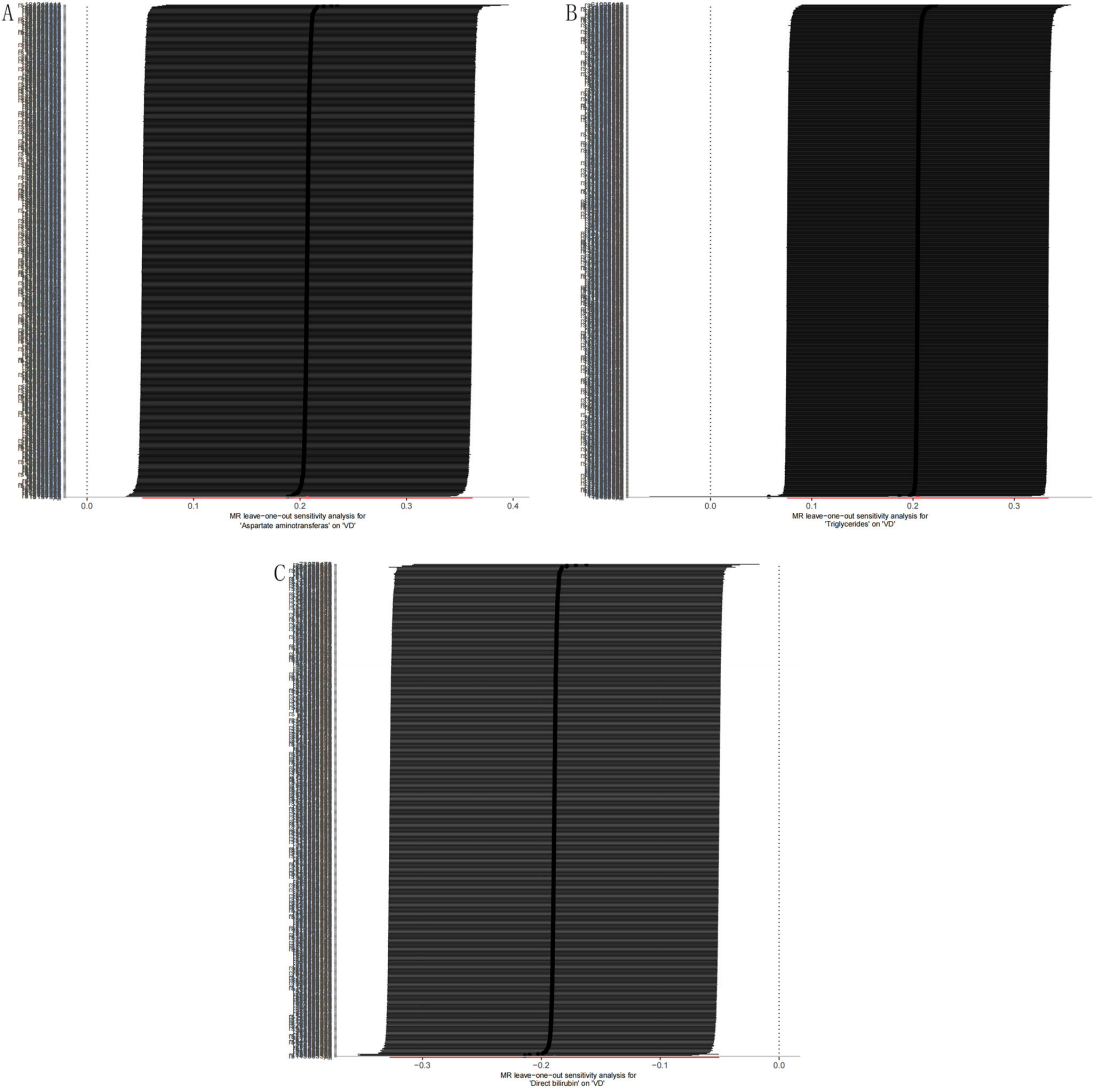
Sensitivity analysis result of the association between AST (A), TG (B), and DBil (C) on VD.

A leave‐one‐out sensitivity analysis was conducted by individually removing each SNP and comparing the causal effects of the remaining SNPs to the overall MR analysis to assess if the causal link was driven by any singular IV. The results from this sensitivity analysis affirmed the robustness of the MR findings (Figure 3).

### 3.2 Causal Influence of VD on 35 Blood and Urine Biomarkers

Following the IV selection framework, VD, considered as an exposure factor, was represented by 17 SNPs, each with an *F*‐statistic greater than 10, indicating a reduced likelihood of bias from weak IVs in the analysis. The reverse MR analysis examining the causal effect of VD on the 35 blood and urine biomarkers showed that VD had no causal impact on these biomarkers (*p* > 0.05).

## 4 Discussion

As the world grapples with an aging population, the sharply rising incidence of cognitive impairment‐related conditions such as VD has significantly strained healthcare systems (Inoue et al. 2023). The prevention, diagnosis, and treatment of VD are fraught with challenges due to its heterogeneous and complex pathology. The cost‐effectiveness and accessibility of blood and urine biomarkers have sparked interest in their potential role as indicators for chronic conditions.

This study embarked on a pioneering investigation into the causal relationships between 35 diverse blood and urine biomarkers and the risk of VD using genetic instruments, facilitated by MR analysis with extensive genetic data. Our integrated findings from both forward and reverse MR analyses revealed that elevated levels of AST and TG are causally linked to an increased risk of VD. Higher levels of DBil are associated with a decreased risk. However, there was no evidence suggesting that genetically predicted VD causally affects these 35 biomarkers. This indicates that AST, DBil, and TG may serve as early predictors for the onset of VD rather than as prognostic biomarkers.

The association between blood and urine biomarkers and VD has been a subject of debate. Previous research, such as a cohort study from the UKB, has examined the relationship between lipid‐related proteins and neurodegenerative diseases including Alzheimer's disease and VD (Gong et al. 2022). That study linked the highest quartile of ApoA levels with a reduced dementia risk, whereas the highest quartile of ApoB levels correlated with an increased risk. According to reports, a study tested the venous blood of 43 patients with vitamin D deficiency and 45 control subjects. It found significant decreases in apolipoprotein A1 and A2 levels in the vitamin D–deficient group (Kuriyama et al. 1994). Additionally, a prospective cohort study on elderly women aged 70–79 found no correlation between C‐reactive protein levels and declines in cognitive and memory functions (Palta et al. 2015). Meanwhile, another longitudinal study reported that higher inflammation levels, as indicated by C‐reactive protein, correlated with lower cognitive function in older adults (Alley et al. 2008). Our MR analysis initially suggested causal links between ApoA, ApoB adjstatins, and C reactive protein with VD (IVW: *p* < 0.05); however, these findings were deemed unreliable upon further pleiotropy testing. This testing indicated potential influences of some IVs on the outcomes through alternative pathways, not directly related to the exposure. Thus, observational studies' results could be confounded. The reverse MR findings further indicated that VD does not affect these biomarkers, reinforcing that there exists no causal link between ApoA, ApoB adjstatins, and C reactive protein and VD.

AST is an enzyme located in the liver, heart, and muscles, playing a crucial role in amino acid metabolism and protein synthesis. According to a prospective cohort study of 431,699 adults, elevated levels of AST and other indicators of liver dysfunction emerged as significant predictors for the onset of all‐cause dementia, Alzheimer's disease, and VD (Gao et al. 2024). This finding aligns with our study, which links increased AST levels to a heightened risk of VD. Interestingly, the same study reported a direct correlation between blood levels of DBil and cognitive decline. However, our MR analysis presents a contrary view, suggesting an inverse relationship between DBil levels and the risk of VD, positing DBil as a potential protective factor in the early stages of the disease. TG, known for their role in lipid metabolism, function as essential markers for energy storage and transport. Research by Christelle Raffaitin and colleagues in a multicenter prospective epidemiological study identified high triglyceride levels as a notable risk factor for developing VD (Raffaitin et al. 2009). Differing perspectives emerge from another cohort study, indicating higher TG levels are linked with increased dementia risk in individuals under 60, but suggesting the opposite effect in those aged 60 and above (Gong et al. 2022). A review and meta‐analysis by Kaarin J Anstey and colleagues reported no association between TG levels in the elderly and an increased risk of VD (Anstey et al. 2017). Our results lend support to the observations made by Christelle Raffaitin, associating genetically predicted increases in TG levels with an elevated risk of VD.

In an aging society, there is an intensified focus on diseases that affect the health and quality of life of the elderly, such as VD (Yuan et al. 2023). The quest for early detection of VD is hampered by the current lack of objective and effective biomarkers. This study represents the first comprehensive investigation into the genetic predictors of 35 different blood and urine biomarkers and their association with VD, employing a bidirectional two‐sample MR approach to minimize confounding and reverse causation biases effectively. The strength of the results from this analysis bolsters our causal conclusions, offering a novel approach to identifying early predictors for VD development. Nonetheless, it is crucial to recognize the limitations of this study, notably that all data were derived from European populations, leaving the applicability of these findings to other populations an open question for further research. Confirming these findings through future clinical trials will be essential.

## 5 Conclusion

Utilizing MR, our study assessed the causal links between various blood and urine biomarkers and VD, identifying AST, TG, and DBil as having causal associations with the development of VD. These insights could facilitate the discovery of biochemical markers for VD prediction, screening, and early diagnosis. Furthermore, therapeutics targeting AST, TG, and DBil might offer promising avenues for effective VD management.

## Author Contributions

**Xiaomin Zhu**: conceptualization, writing – original draft, writing – review and editing. **Yanan Hu**: software. **Jieshuang Lin**: formal analysis. **Guifeng Zhuo**: software, formal analysis, writing – review and editing. **Yingrui Huang**: formal analysis. **Yulan Fu**: formal analysis. **Ying Zhang**: Validation. **Lin Wu**: Funding acquisition, writing – review and editing. **Wei Chen**: Funding acquisition, writing – review and editing.

## Conflicts of Interest

The authors declare no conflicts of interest.

## Peer Review

The peer review history for this article is available at https://publons.com/publon/10.1002/brb3.70736.

## Data Availability

Research data are not shared.
